# Distance education during COVID 19: an Italian survey on the university teachers’ perspectives and their emotional conditions

**DOI:** 10.1186/s12909-021-02780-y

**Published:** 2021-06-09

**Authors:** Massimo Casacchia, Maria Grazia Cifone, Laura Giusti, Leila Fabiani, Roberto Gatto, Loreto Lancia, Benedetta Cinque, Cristina Petrucci, Mario Giannoni, Rodolfo Ippoliti, Anna Rita Frattaroli, Guido Macchiarelli, Rita Roncone

**Affiliations:** Department of Life, Health and Environmental Science, University of L’Aquila, L’Aquila, Italy

**Keywords:** Distance education, COVID-19 pandemic, University, University teachers, Medical courses

## Abstract

**Background:**

Following the COVID-19 pandemic, distance education (DE) replaced traditional “face-to-face” teaching and has become the main method of teaching. The aim of this study was to 1) evaluate the impact of DE by teachers in our department during the second semester of the 2019–20 academic year following the March–May 2020 Italian national lockdown and 2) evaluate the relationship between DE and the emotional well-being of teachers during the period of home confinement.

**Methods:**

Ninety-seven university teachers (51.5% women; most represented age group 60–69 years range, 40.2%) responded to an anonymous online cross-sectional survey between July 15 – September 30, 2020, on the advantages and disadvantages of DE, developed by one online teacher focus group. The emotional conditions were assessed by a short version of the Beck Depression Inventory-II (BDI-II). The internal consistency reliability survey and the 10-item BDI-II were measured by Cronbach’s alpha. A correlation analysis (r-Pearson) was conducted between the overall evaluation of the experience of DE and the variables included in the study.

**Results:**

Teachers reported difficulties in technical aspects, and in psychological factors, as the discomfort of “speaking in the void” (64.7%). The absence of “face-to-face” eye contact with the students was complained by 81% of teachers.

Significant impairments in sleep patterns and loss of energy were reported, with female teachers having greater difficulty concentrating than their male colleagues. A quarter of teachers showed depressive symptoms of varying severity.

The most satisfied teachers were those most stimulated by DE (r = 0.752, *p* < 0.000), who showed a lower impact of depressive symptoms (r = − 0.289, *p* = 0.005). The teaching load in hours influenced the perception of disadvantages (r = 0.214, *p* = 0.035) and contributed to a lower appreciation of the challenges of DE.

The more significant the manifestation of depressive symptoms during the lockdown was, the greater the subjective recovery of a good emotional condition once the domestic confinement was over (r = 0.344, *p* = 0.001), despite maintaining DE.

**Conclusions:**

Our study highlights the impact of technical, didactic, and psychological difficulties of DE, reported by our teachers. The appreciation of their new learning promoted by DE seemed related to better emotional well-being of university teachers accepting this “challenge” in their important role in the high-education system, influencing good learning and promoting students’ professional success.

**Supplementary Information:**

The online version contains supplementary material available at 10.1186/s12909-021-02780-y.

## Background

Since the beginning of 2020, Europe has been hit by the sudden and terrible global epidemic of COVID-19 [1]. On March 10, 2020, Italy was placed in total lockdown (“phase 1”). The security measures adopted to contain the pandemic, which were maintained in rigorous form until May 4, 2020 (the beginning of “phase 2”), have led to significant and persistent changes in the way we live, work, and study. Domestic confinement was adopted, and many productive activities and “face-to-face” training activities in primary and secondary schools and universities were interrupted.

In the educational field, there are many challenges that all universities have faced and are still facing [2], such as changing the teaching methods from “face-to-face” to conducting online lessons, developing valid methods for verifying learning with particular reference to the performance of written tests, addressing the problem of internships and professional activities, and addressing the problem of internationalization in light of the containment measures of the COVID-19 epidemic, which entail restrictions on mobility between different countries and within a country.

Distance education (DE) has replaced the traditional method of face-to-face teaching and has become the main method of teaching. DE is a planned learning activity done by individuals in different places, who communicate and interact with each other by using technological tools [3]. The “sudden” and massive use of technologies resulting from the social distancing imposed by the COVID-19 pandemic required great flexibility from students and university professors [2]. Within a few days and despite difficulty procuring goods and services, the latter had to radically change their teaching methodologies to use IT tools and digital platforms.

In organizations, the use of information and communication technology (ICT) has always been a topic involving many controversies and paradoxes [4]. With the gradual digitization of university education, which is very slow in our system, ICT has undoubtedly brought benefits to university teachers’ work. ICTs such as mobile computing, collaborative software, and the teaching management system allow teachers to work from anywhere and at any time and to conveniently access information and updates [5]. However, ICTs also pose challenges to people’s physical and psychological well-being and job performance [6]. For example, ICTs can push academics to work more intensively than they can handle (technological overload) and invade their personal life (technological invasion). Frequent changes and updates of software and hardware can make university teachers feel as if they are not very competent (technological complexity and technological uncertainty), with the frequent perception of the loss of security of their systems (techno-insecurity). As a result, academics may feel exhausted, anxious, and stressed. This phenomenon has been defined as “technostress” [7], and the core assumption of technostress literature is that ICT may be perceived as stressful [8]. Technostress was associated with psychological and behavioral disorders and impairment of job and life satisfaction, leading to reduced productivity [9, 10]. Technostress seems to influence university teachers’ work [11, 12] and has a more significant impact on older university teachers than on younger ones [13].

Finally, the impact of the COVID-19 pandemic on the general population’s mental health must be considered [14, 15]. This impact is also relevant to the university student population [16, 17] and the entire academic population. Among the factors related to an increase in anxiety symptoms, delays and/or slowdowns in academic activities in the early stages of the pandemic have been highlighted [16]. A university survey conducted in Spain found higher levels of anxious and depressive symptoms in students than university staff (academic and administrative), suggesting that students have suffered more from the psychological impact of the current health emergency of COVID-19 [18].

A very recent study conducted in Jordan assessed in what way the COVID-19 pandemic and the resultant quarantine/national measures impacted the psychological status of university teachers and their perceived motivation, challenges, and technostress due to distance teaching strategy along with their self-adaptive activities during this pandemic [19]. The Authors reported various levels of psychological distress among approximately the 70% of academic staff highly concerned and fearful about SARS-CoV-2 infection, social isolation, as well as the economic impact of the pandemic. They showed a good attitude toward distance/remote teaching, as 72.8% had moderate to high motivation toward this educational strategy. Meanwhile, they we were worried about the increased likelihood of cheating among students during online examinations, the efforts and time needed to prepare online examinations and fair evaluation assignments, the intrusion of privacy, and reduced interaction with students [19].

In Italy, little attention was paid to university teachers and their DE experience, which was introduced quickly and posed a real challenge to the pedagogical content knowledge (PCK) needed to teach online [20], and to their emotional well-being. The fear of COVID-19, the home-confinement, and the strict control measures, as well as social isolation that were forced during the current pandemic, could all result in the deterioration of the psychological status of various social components, including academicians [19]. They play a crucial role with university institution in designing effective learning environments for faculty professional development [21]. Not paying attention to teachers, an underrepresented group, and their difficulties could influence the quality of their didactic also and the learning and future professional success of their students [22].

From this perspective, the aim of our study was to 1) evaluate the impact of DE by teachers in our department during the second semester of the 2019–20 academic year following the March–May 2020 Italian national lockdown and 2) evaluate the relationship between DE and the emotional well-being of teachers during the period of home confinement. We expected that the organizational changes imposed by DE and the related challenges could have had a negative impact on the psychological well-being of teachers during the Italian lockdown in a global climate characterized by the fear of COVID-19 contagion and uncertainty.

## Methods

### Study design and participants

We conducted a cross-sectional online anonymous survey design using a convenience sample in the period July 15 – September 30, 2020, before the opening of the new academic year (A.Y.) 2020–21.

On July 15, 2020, the survey on DE addressed to the department’s teachers was loaded on the department’s home page through advertising “Teaching with COVID”. All teachers involved in the department degree courses received an invitation to participate in the survey by e-mail through a link to a “Microsoft Forms” form on the department’s page. The participants did not receive any form of compensation for participation in this study.

### Context

In L’Aquila’s context, the health emergency of COVID-19 and related academic-organizational changes probably represented an additional challenge that teachers had to face after the devasting earthquake that hit L’Aquila on April 6, 2009, bringing death and destruction to the University of L’Aquila, with 55 students killed [23–25]. Some of the university staff restarted their activities 3 days after the earthquake, but some damaged university buildings’ reconstruction process is still ongoing. This catastrophic event meant that most of the University teachers currently in service had already faced emergencies. Some are still in temporary accommodation with their clinical activities and laboratories, which implies a persistent stressful condition.

The Department of Life, Health and Environmental Science of the University of L’Aquila manages 17 study programs (7 master’s degree courses and 10 degree courses) in 3 areas - medical, biological, and environmental sciences - with 2773 students enrolled in 2019 and with a teaching staff of 125 tenured teachers. Starting on March 13, 2020, the University of L’Aquila made it possible to start DE through the use of the Teams - Microsoft platform, with extensive communication via e-mail of the procedures for its use and availability advice from the Sector for E-Learning and Advanced Teaching.

### Survey instrument and related measures

Microsoft Form® (Microsoft Corporation, Redmond, Washington, USA) was used to create the online survey, “Teaching with COVID”.

The survey represented the results of one online focus group planned on Microsoft Teams® (Microsoft Corporation, Redmond, Washington, USA) to develop concepts and questions for the questionnaire design. The focus group meeting lasted 2 h and included teachers involved in the Department’s Quality Working Group (QWG). Implemented to coordinate the Quality Policy’s actions, the QWG was established in October 2019, consisting of professors related to the degree courses. The QWG works in cooperation with the department’s Teacher-Student Joint Commission and its president, MG C.

The focus group identified 3 main issues related to their DE experience: technological, teaching, and psychological aspects based on teachers’ contributions, their main appreciation, and complaining about the work done during the lockdown with their students. The psychiatrists (MC, RR) and the clinical psychologist (LG) suggested including two validated instruments to assess the psychiatric symptoms. After a long discussion, the QWG decided to add only a short version of the Beck Depression Inventory-II, BDI-II [26], since most of the focus group participants did not agree in “psychiatrize” what could be defined as “normal reactions” to the unusual and stressful condition of the COVID-19 mass home confinement. Ten items of the BDI-II were selected and included in an optional third section to avoid that the survey was too invasive and long in filling out.

Five full professors (not involved in the QWG focus group that implemented the survey) were asked to join a piloting phase to assess the survey’s face validity, phrasing, and clarity. This piloting phase resulted in a minor modification of the initial version of the survey (linguistic change to clarify ten answers better). The responses from the piloting step were not included in our analysis.

Three main sections were included in this anonymous online survey (see in Additional file 1: Appendix 1). The form included closed-ended questions with a limited set of possible answers. The only open-ended question was asked to ask “Comments and suggestions” at the end of the second section.

The first preliminary section concerned information on the study and on the protection of privacy, ensuring total confidentiality of the personal data as also provided by 181 the European General Data Protection Regulation n. 2016/679. The lack of consent did not allow the compilation of the form.

The second section included six sub-sections (see Additional file 1: Appendix 1). The first subsection collected the main demographic and academic data, in the period considered (second semester A.Y. 2019–20). The second subsection investigated the experience in the delivery of the teaching module through DE.. The third subsection explored the advantages of conducting DE.. Also, satisfaction regarding new personal learning due to the use of digital tools was assessed in this sub-section. The fourth subsection explored the disadvantages in conducting the didactic module through DE.. The fifth subsection investigated the “colleagueship”, i.e., the support provided to colleagues in difficulty/support received by colleagues. The sixth subsection include the overall evaluation of the DE experience, for which the teachers were asked for an overall evaluation on a 10-point Likert scale (1 = not satisfied at all; 10 = very satisfied).

The third section provided for an optional compilation. It was centered on the teacher’s emotional well-being during COVID-19 lockdown and included a 10-item short version of the Beck Depression Inventory-II (BDI-II) [26]. The BDI-II is a 21-item inventory that measures the severity of self-reported depression over the prior 2 weeks; its item content corresponds to criteria for diagnosing depressive disorders as specified in the Diagnostic and Statistical Manual of Mental Disorders-IV (DSM-IV). Items are structured on a 4-point scale ranging from zero points (symptom not present) to three points (symptoms strongly present). Thus, a BDI-II total score from 0 to 13 points represents normal to minimal depression, 14 to 19 points indicates mild depression, 20 to 28 points indicates moderate depression, and 29 to 63 points indicates severe depression. For the present study, 10 items were selected to facilitate the compilation of the tool (see Additional file 1: Appendix 1). In our 10-item version of the scale, we considered as a cut-off a score of 6, with scores in the range of 6–8 = mild depression, 9–12 = moderate depression, and 13–27 = severe depression.

In the end, each teacher was asked for an evaluation of their perceived emotional condition after the period of confinement (see Additional file 1: Appendix 1).

University teachers were invited to participate in the study through corporate email.

### Statistical analysis

The internal consistency reliability survey and the 10-item BDI-II were measured by Cronbach’s alpha. Parametric and nonparametric tests were used for data analysis. The t-test for independent samples and the chi-square test were used to examine the differences in sociodemographic variables in the variables related to the investigated aspects of DE in teachers, such as age, gender, and university role.

A correlation analysis (r-Pearson) was conducted to verify relationships among the overall evaluation of the DE experience, the age of the teachers, teaching hours in the second semester 2020, the number of advantages and disadvantages of DE, satisfaction with new learning due to the use of digital tools, the scores reported by teachers on the BDI-II during the lockdown, and the evaluation of emotional health after the confinement period. Statistical analyses were conducted using SPSS 26.0 (SPSS Inc., Chicago, IL, USA).

## Results

### Demographic and academic data

The main characteristics of the participants are shown in Table 1.

**Table 1:** Main characteristics of the participants (*n* = 97)

Socio-academic characteristics	Total (%)
**Age range, n (%)**	
< 39 years	6 (6.2)
40–49 years	17 (17.5)
50–59 years	31 (32)
60–69 years	39 (40,2)
> 70 years	4 (4.1)
**Sex (%)**	
Men	47 (48.5)
Women	50 (51.5)
**Academic role, n (%)**	
Tenured teachers:	
• Full professor	23 (23.7)
• Associate professor	24 (24.7)
• Researcher	17 (17.5)
• “Temporary” researcher	11 (11.4)
Contract teachers	15 (15.5)
Hospital contract teachers	7 (7.2)
**First- and second-level degree courses where the teachers taught, n (%)**	
Second-Level Degree (unique 6-year cycle) Course in Medicine and Surgery	40 (41.2)
Second-Level Degree (unique 6-year cycle) in Dentistry and Dental Prosthetics	20 (20.6)
Second-Level Degree in Nursing and Obstetric Sciences	8 (8.2)
Second-Level Degree in Sciences of Technical Health Professions	13 (13.4)
Second-Level Degree in Sciences of Prevention Health Professions	10 (10.3)
First-Level Degree Course in Nursing	13 (13.4)
First-Level Degree Course in Speech Therapy	13 (13.4)
First-Level Degree Course in Obstetrics	12 (12.4)
First-Level Degree Course in Orthoptics and Ophthalmological Assistance	8 (8.2)
First-Level Degree Course in Psychiatric Rehabilitation Technique	17 (17.5)
First-Level Degree Course in Neuro and Psychomotor Childhood Therapy	20 (20.6)
First-Level Degree Course in Dental Hygiene	19 (19.6)
First-Level Degree Course in Techniques of Prevention in the Environment and in the Workplace	10 (10.3)
Second-Level Degree Course in Biology of Health and Nutrition	10 (10.3)
First-Level Degree Course in Biological Sciences	11 (11.3)
Second-Level Degree Course in Environmental Biology and Ecosystem Management	7 (7.2)
First-Level Degree Course in Environmental Sciences and Technologies	4 (4.1)
**Teachers who held lessons in the second semester academic year 2019–20, n (%)**	
One course	37 (38.1)
More courses	48 (49.5)
Lessons held in the first semester	12 (12,4)
**Mean teaching hours in the second semester, mean (+SD)**	50 (+ 40.8)
**Teachers who also held lessons in the master’s program, n (%)**	23 (23.7)
**Teachers who also held lessons in other departments, n (%)**	26 (26.8)

The questionnaire was completed by 97 teachers with a mean age of 56.13 years (SD 10.5). Women were more represented and younger (mean age: 53.9, SD 10.3) than male teachers (58.4, SD 10.2) (t-test for independent samples: 2.183; *p* = 0.031). The most represented age group was in the range of 60–69 years (40.2%).

The tenured teachers who completed the survey (*n* = 75, 77.3% of our sample) represented 60% of our department’s full-time teacher population. Alongside the tenured teachers, 15 contract teachers and 7 teachers of the health professions (“hospital contract teachers” working within the Local Health Unit ASL 1 Avezzano-Sulmona L’Aquila of the Abruzzo Region) responded to the survey (22.7%). The majority of teachers who responded were those who taught in the Course in Medicine and Surgery, and the majority were associate professors. More than 85% of teachers (*n* = 85) taught in the second semester of the academic year 2019–2020, and almost 50% had several courses with an average teaching load of 50 h (range 10–240). Approximately one-quarter of them also conducted lessons in the master’s degree and in other departments. The 12 teachers (12.4%) who had not taught referred to the online exam sessions and completed only the relevant items. Internal consistency for the questionnaire was high in the sample for the whole measure (Cronbach’s α = 0.854) and within the second and third sections (second section α = 0.833; third section α = 0.813).

### The experience of delivery of the didactic module through DE

#### Technological aspects

Among the 85 teachers who conducted their lessons in the second semester, teachers reported the quality of connections as the most problematic issue (Table 2). Furthermore, approximately 50% reported difficulties in using the Teams institutional platform. Overall, of the 3 items considered, 16.5% reported at least one very problematic aspect. In the household, overlapping problems using the network and hardware were reported by 43.5% of teachers, in 9.4% of cases with significant inconvenience and frequent loss of effectiveness of connections and/or alternation in hardware sharing.

**Table 2:** Technical and didactic aspects of distance education (*n* = 85)

Technical aspects of distance education	Total (%)
**1. Connection quality**	
No difficulties	23 (27.1)
Occasional difficulties	40 (47.0)
Some difficulties	12 (14.1)
Many difficulties	10 (11.8
Major difficulties	–
**2. Device availability**	
No difficulties	61 (71.7)
Occasional difficulties	17 (20)
Some difficulties	6 (7.1)
Many difficulties	1 (1.2)
Major difficulties	–
**3. Use of the Teams platform**	
No difficulties	45 (52.9)
Occasional difficulties	31 (36.5)
Some difficulties	5 (5.9)
Many difficulties	4 (4.7)
Major difficulties	–
**4. Family overlap problems in the use of networks and hardware**	
No problems	48 (56.5)
Minor inconveniences, without significant consequences	29 (34.1)
Significant inconvenience and frequent loss of effectiveness of the connection	8 (9.4)
Frequent inability to connect due to network or hardware sharing	–
**Didactic aspects of distance education**	
**1. Work-time materials for DE**	
No difficulties	23 (27.1)
Occasional difficulties	18 (21.1)
Some difficulties	29 (34.1)
Many difficulties	10 (11.8)
Major difficulties	5 (5.9)
**2. Work-time recording lessons for DE**	
No difficulties	41 (48.2)
Occasional difficulties	14 (16.5)
Some difficulties	22 (25.9)
Many difficulties	4 (4.7)
Major difficulties	4 (4.7)
**3. Supervision of oral examinations**	
No difficulties	43 (50.6)
Occasional difficulties	12 (14.1)
Some difficulties	13 (15.3)
Many difficulties	12 (14.1)
Major difficulties	5 (5.9)
**4. Supervision of written examinations**	
No difficulties	36 (42.4)
Occasional difficulties	14 (16.5)
Some difficulties	10 (11.8)
Many difficulties	16 (18.8)
Major difficulties	9 (10.5)

#### Didactic aspects - working time and complexity of learning assessments in DE

In our sample of teachers, the most problematic areas in the delivery of DE seemed to be represented by greater time-work for the organization, greater commitment to structuring the materials for the DE lessons, and greater effort in the supervision required in conducting remote exams, especially written assignments (Table 2). Almost a fifth of the teachers (18 teachers, 21.2%) reported scores above the cut-off of 8 on the 4 items relating to the didactic aspects in conducting DE, highlighting significant difficulties in this area.

### Psychological aspects of DE

In this section, 64.7% of teacher complained about the *“discomfort of speaking” in the void “through a camera, without face-to-face contact with the students”,* and among them almost a quarter of the teachers reported the greatest and most relevant difficulties (many difficulty – major difficulties = 24.7%) (Table 3). A few teachers perceived a sense of intrusion into their domestic privacy due to providing lessons from their home. Considering those who exceeded the total score of 12 in the 6 items relating to this area, highlighting greater difficulties in this area, 16 teachers (18.8%) reported scores higher than this cut-off.

**Table 3:** Psychological aspects of distance education (*n* = 85)

Psychological aspects of distance education	Total (%)
**1. Speaking “in the void”**	
No difficulties	30 (35.3)
Occasional difficulties	17 (20)
Some difficulties	17 (20)
Many difficulties	13 (15.3)
Major difficulties	8 (9.4)
**2. Accuracy of materials to be left to students**	
No difficulties	39 (45.9)
Occasional difficulties	16 (18.8)
Some difficulties	17 (20)
Many difficulties	10 (11.8)
Major difficulties	3 (3.5)
**3. Acritical diffusion of uploaded materials on TEAMS by the students**	
No difficulties	28 (32.9)
Occasional difficulties	16 (18.8)
Some difficulties	18 (21.2)
Many difficulties	23 (27.1)
Major difficulties	–
**4. Lack of interest in online lessons by students**	
No difficulties	25 (29.5)
Occasional difficulties	20 (23.5)
Some difficulties	24 (28.2)
Many difficulties	16 (18.8)
Major difficulties	–
**5. Be recorded during lessons**	
No difficulties	47 (55.3)
Occasional difficulties	10 (11.8)
Some difficulties	18 (21.2)
Many difficulties	10 (11.8)
Major difficulties	–
**6. Be observed in one’s own private household**	
No difficulties	65 (76.5)
Occasional difficulties	9 (10.6)
Some difficulties	7 (8.2)
Many difficulties	4 (4.7)
Major difficulties	–

### Advantages of DE

#### Relationship with students

**Fig. 1:**
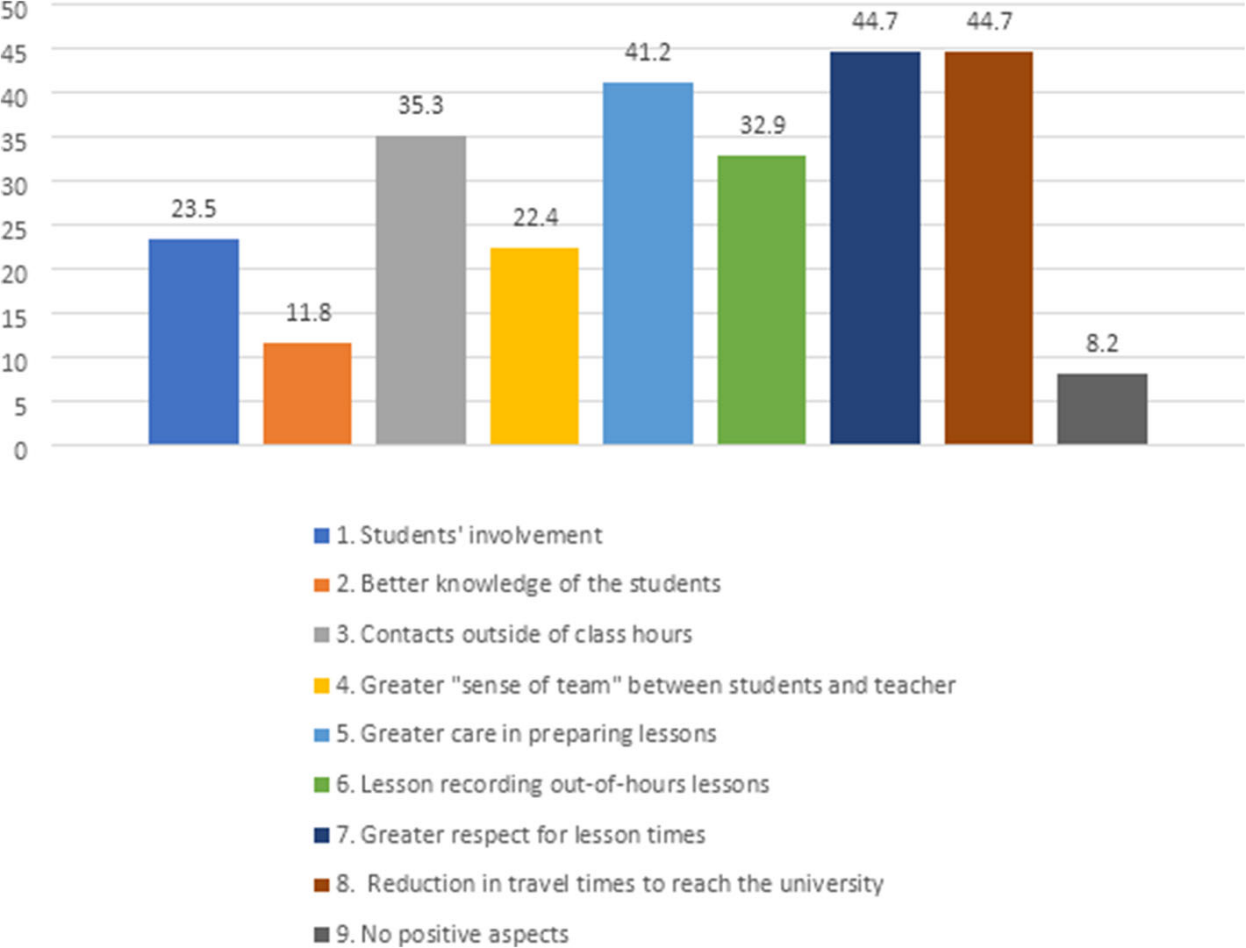
Advantages of distance education (%)- Relationship with students (1–4 item/bars)– Didactic and organizational aspects (5–8 item/bars) (*n* = 85)

Among the 85 teachers who conducted their lessons in the second semester, only 8 (8.2%) reported that they did not find any positive aspects of DE (Fig. 1). The use of digital media allowed a greater opportunity for contact outside of class hours (via e-mail or WhatsApp) (*n* = 30, 35.3%). A little more a quarter of teachers perceived a good students’ involvement (*n* = 20, 23,5%). A total of 22.4% (*n* = 19) of teachers reported that they appreciated a greater “sense of team” between students and teachers aimed at preparing for exams in conditions of perceived “emergency.” Additionally, a higher number of attending students was reported.

#### Didactic and organizational aspects

Most of the teachers expressed several advantages (multiple answers were possible) in this area, with equal appreciation for the reduction in travel time to reach the university by nonresident teachers (*n* = 38, 44.7%) and greater respect for lesson times (*n* = 38, 44.7%) (Fig. 1). Greater care represented a positive aspect stimulated by DE in preparing the lessons for the teachers (*n* = 35, 41.2%), who tried to identify with the students and to stimulate and involve them, even at a distance.

#### Savings

In the period of confinement, regarding the provision of DE, 70.6% (*n* = 60) of teachers managed to “save”. This number mainly involved teachers who were not resident in the city of L’Aquila due to the missed fuel expenses to reach the workplace, catering costs and any overnight stays at the workplace, with an average savings of 529 euros (SD + 712; range 20–3000 euros). The 29.4% of teachers who “failed to save” in the period considered included clinicians, who had to reach the hospitals’ wards at their respective locations.

#### Exchange opportunities with colleagues

Including all sample of 97 teachers, in consideration of their involvement in the academic life, 69% of teachers (*n* = 67) reported having experienced opportunities to improve exchanges with colleagues during the second semester AY 2019–20, while the remaining 31% (*n* = 30) did not report this opportunity.

Thirty-one percent of the teachers (*n* = 31) appreciated better coordination with the other teachers of the Integrated Course to agree on the examination methods, and 16 teachers (16.5%) reported greater opportunities for discussion among teachers promoted by the Integrated Course Coordinator. Only 4 teachers (4.1%) reported to have been in contact with the semester coordinators of courses.

#### New individual learning

Among the 85 teachers who conducted their lessons in the second semester, with the exception of 8 teachers (9.4%) who reported that they had not learned anything new, more than 90% of teachers appreciated the improvements in their digital skills.

A total of 24.7% of teachers (*n* = 21) reported learning from scratch on digital platforms and/or improvement in their use. Very similar percentages of teachers (23.5%, *n* = 20) reported improvements in PowerPoint lecture presentations (e.g., animations, video file insertion, transformation into.mp4 files) and learning from scratch and/or improving the use of platforms/software for conducting online exams (20%, *n* = 17). A smaller percentage (12.9%, *n* = 11) of teachers reported improvements and/or learning from scratch on platforms and specific software for teaching in the related subject areas. Regarding the self-assessment of teachers’ satisfaction with their personal improvements in computer and digital skills, the average score was 6.63 (SD + 2.18).

### Disadvantages of DE

#### Relationship with students

**Fig. 2:**
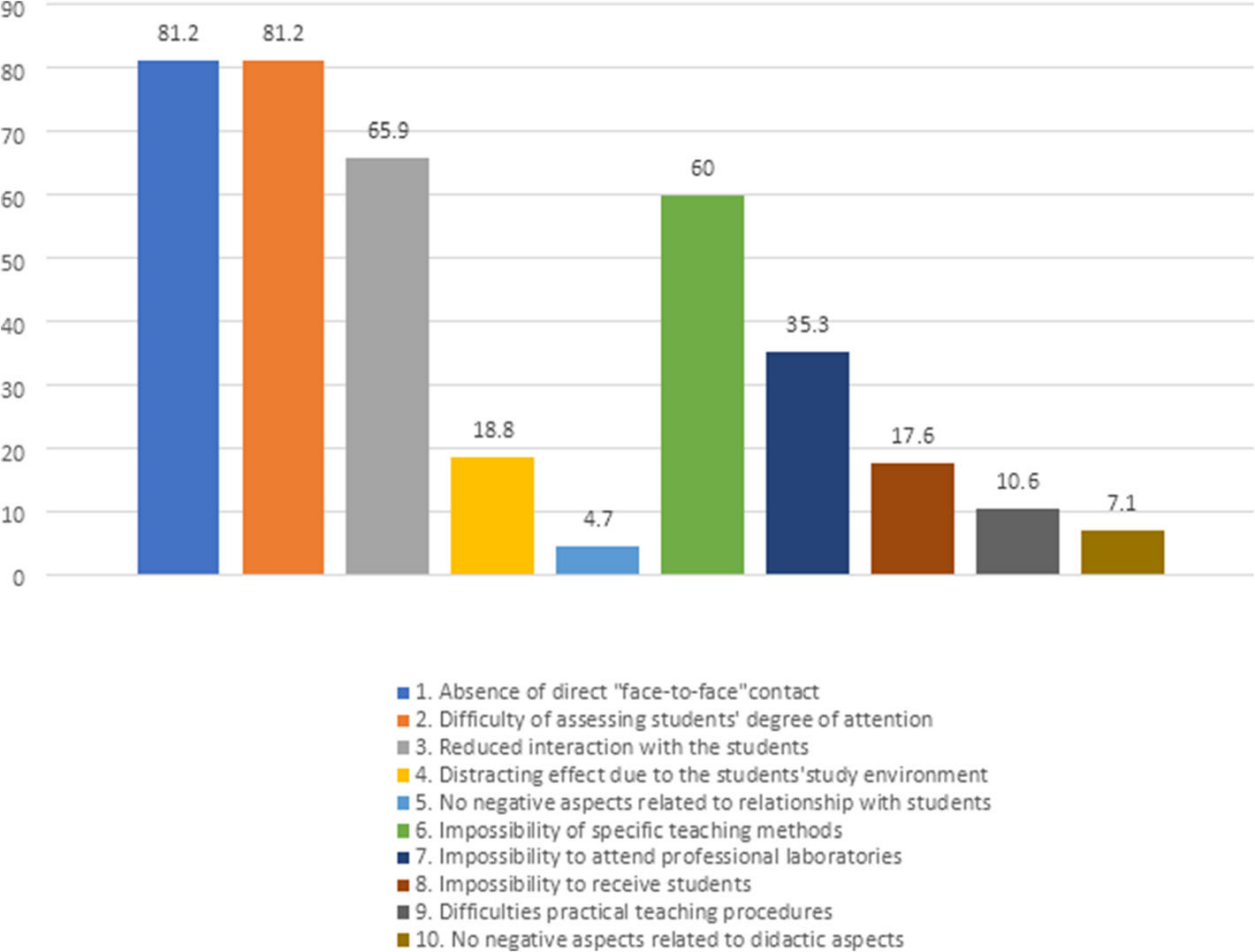
Disadvantages of distance education (%)- Relationship with students (1–5 item/bars)– Didactic and organizational aspects (6–10 item/bars) (*n* = 85)

Only 4 teachers (4.7%) in our sample who taught in the second semester AY 2019–20 did not find any disadvantages of DE with regard to the relationship with students (Fig. 2). Most of the teachers (*n* = 69, 81,2%) complained about the difficulty of assessing students’ degree of attention because of the absence of direct “face-to-face” eye contact. Complaints about reduced interaction with students during lessons were expressed by 65.9% of teachers (*n* = 56), and 18.8% (*n* = 16) reported a distracting effect due to the students’ study environment.

#### Didactic and organizational aspects

Six teachers (7.1%) found no disadvantages of DE with regard to the didactic and organizational aspects of online delivery (Fig. 2). The impossibility of conducting specific teaching methods (e.g., work in small groups, role play) was highlighted by 60% of the teachers (*n* = 51). The impossibility of organizing laboratory professionalizing activities was noted by 35.3% of teachers (*n* = 30), especially by professors of medical and health professions courses. The difficulty of receiving students was reported by 17.6% of teachers (*n* = 15), while 10.6% (*n* = 9) reported difficulties related to carrying out even small procedures related to teaching.

#### Expenses incurred

A total of 45.9% of teachers (*n* = 39) incurred additional expenses in conducting DE from their home due to telephone and network consumption, the purchase of headphones and microphones, consumables (e.g., toner, cartridges, paper), and the purchase of personal computers, with an average cost of 348 euros (SD + 492; range 20–2000 euros).

### Support provided and received from colleagues in conducting distance education

More than half of the sample (54.1%) reported that they helped colleagues who had requested help, whereas 39 teachers (45.9%) did not receive any request (Table 4). The support provided was mainly about the practical aspects of conducting teaching on the Teams platform.

**Table 4:** Support provided and received in conducting distance education (*n* = 85)

Support provided	Total (%)
I helped colleagues in the new planning of teaching activities	15 (17.6)
I helped my colleagues with the practical aspects related to the Teams platform	38 (47.0)
I helped colleagues in carrying out the DE activity	14 (16.5)
I helped Colleagues in maintaining contact with other teachers	3 (3.5)
I helped Colleagues in maintaining contact with students	2 (2.4)
I did not received any request	39 (45.9)
**Support received**	
I was helped by my colleagues in the new planning of the teaching activity	18 (21.2)
I was supported by my colleagues with the practical aspects related to the Teams platform	44 (51.8)
My colleagues helped me in carrying out the DE activity	15 (17.6)
My colleagues helped me in maintaining contact with other teachers	4 (4.7)
I was assisted by my colleagues in maintaining contact with students	3 (3.5)
Although I wanted to be helped, I found no support	2 (2.4)
Other (University’s IT service assisted with the practical aspects related to the Teams platform)	10 (11.8)
I didn’t need any support	30 (35.3)

Approximately a quarter of our sample (35.3%) reported that they did not need any support in DE provision and had used the instructions sent by email provided by the university’s IT service, which was also available by telephone. Ten teachers (11.8%) reported that they did not ask their colleagues and addressed their requests to the University’s IT service. More than 50% of the sample (*n* = 44) reported having been helped by colleagues in conducting teaching activities related to the practical aspects of the Teams platform. Only 2 (2.4%) of the teachers participating in the study said they were dissatisfied with the support received and/or dissatisfied with their expectations.

### Overall evaluation of the experience of DE

More than 50% of teachers scored higher than 6 (Table 5). The average teacher evaluation score assigned to the DE experience was 6.32 (SD + 2.19; range-1-10), with a sufficient overall score.

**Table 5:** Overall evaluation of the experience of DE (*n* = 97)

Evaluation scores	Total (%)
1–3	8 (8.3)
4–5	28 (28.7)
6	8 (8.3)
7–8	37 (38.2)
9–10	16 (16.5)

### Suggestions

The only open-ended question was related to suggestions and comments about DE at the end of the second section. Including the sample of 97 teachers, 15 teachers (15.5%) gave their contribution and commented. In summary, the teachers reported that DE represented an alternative modality valid in emergencies. They stressed that in normal conditions, it could be integrated with frontal teaching, after programming, and training, not only of a strictly technical nature on the use of platforms. Some teachers stressed that direct interaction with students remains “indispensable” along with the need to stay on the field for internships and laboratory experiences, impossible with DE, reiterating the importance of maintaining clear distinction with telematic universities. Only one teacher expressed his enthusiasm about DE, reporting the virtual experience better than in the classroom. One teacher described his concern about the need to prevent academic dishonesty in exams by the students.

### Emotional aspects of teachers

Table 6 shows the scores on the 10-item BDI-II of teachers regarding their emotional conditions during the lockdown.

**Table 6:** Emotional impairment of teachers (10 items derived from the BDI-II) referring to the confinement period (*n* = 97)

Sadness	N (%)
0. I did not feel sad	70 (72.2)
1. I felt sad	23 (23.7)
2. I was sad all the time and I couldn’t snap out of it	1 (1)
3. I was so sad or unhappy that I couldn’t stand it	0
Missing	3 (3.1)
**Pessimism**	
0. I was not particularly discouraged about the future	65 (67)
1. I felt discouraged about the future	29 (29.9)
2. I felt I had nothing to look forward to	0
3. I felt the future was hopeless and that things could not improve	0
Missing	3 (3.1)
**Loss of pleasure**	
0. I got as much satisfaction out of things as I used to	71 (73.2)
1. I didn’t enjoy things the way I used to	5 (5.2)
2. I didn’t get real satisfaction out of anything anymore	5 (5.2)
3. I was dissatisfied or bored with everything	13 (13.4)
Missing	3 (3.1)
**Loss of interest**	
0. I didn’t lose interest in other people or activities	86 (88.7)
1. I was less interested in other people or things than before	6 (6.2)
2. I lost most of my interest in other people or things	1 (1)
3. It was hard to get interested in anything	1 (1)
Missing	3 (3.1)
**Loss of energy**	
0. I had as much energy as ever	68 (70.1)
1. I had less energy than I used to have	25 (25.8)
2. I didn’t have enough energy to do very much	1 (1)
3. I didn’t have enough energy to do anything	0
Missing	3 (3.1)
**Changes in sleeping pattern**	
0. I did not experience any change in my sleeping pattern	43 (44.3)
1.a I slept somewhat more than usual	22 (22.7)
1.b I slept somewhat less than usual	20 (20.6)
2.a I slept a lot more than usual	2 (2.1)
2.b I slept a lot less than usual	3 (3.1)
3.a I slept most of the day	0
3.b I woke up 1–2 h early and I couldn’t get back to sleep	4 (4.1)
Missing	3 (3.1)
**Irritability**	
0. I was not more irritable than usual	70 (72.1)
1. I was more irritable than usual	21 (21.6)
2. I was much more irritable than usual	3 (3.1)
3. I was irritable all the time	0
Missing	3 (3.1)
**Changes in appetite**	
0. I did not experience any change in my appetite	66 (68)
1.a My appetite was somewhat less than usual	5 (5.1)
1.b My appetite was somewhat greater than usual	23 (23.5)
2.a My appetite was much less than usual	0
2.b My appetite was much greater than usual	0
3.a I had no appetite at all	0
3.b I craved food all the time	0
Missing	3 (3.1)
**Concentration difficulty**	
0. I could concentrate as well as ever	75 (77.3)
1. I couldn’t concentrate as well as usual	16 (16.5)
2. It was hard to keep my mind on anything for very long	3 (3.1)
3. I found I couldn’t concentrate on anything	0
Missing	3 (3.1)
**Tiredness or fatigue**	
0. I was no more tired or fatigued than usual	60 (61.9)
1. I got tired or fatigued more easily than usual	31 (32)
2. I was too tired or fatigued to do a lot of the things I used to do	3 (3.1)
3. I was too tired or fatigued to do most of the things I used to do	0
Missing	3 (3.1)

The mean total score on the 10 items of the BDI-II (range 0–14) was 3.40 (SD + 3.64), with no difference between genders. Approximately three-quarters of the teaching population had normal scores; 25.8% of teachers exceeded the cut-off for depressive symptomatology, with 15.1% of teachers whose scores could be reported as mild depressive symptomatology, 7.5% moderate depressive symptomatology, and 3.2% severe symptomatology.

Regarding the different items surveyed by the scale, approximately 30% of the sample reported feeling more discouraged than usual about the future. Sixty percent of the sample reported changes in sleep patterns, and nearly 40% of teachers reported more tiredness and fatigue than usual. Almost 30% of teachers reported changes in appetite, with a percentage close to 25% reporting an increase in appetite.

We did not find any statistically significant gender differences, with the exception of the item “*Concentration Difficulty”*, with greater difficulty concentrating among women (0.37, SD + 0.60) than men (0.09, SD + 0.28; t = − 2.821; *p* = 0.006).

Based on the score on the BDI-II, teachers showing depressive symptomatology were not represented to a greater extent among teachers who had attended several courses compared to those who had only taught one or had not taught in the second semester. There were no statistically significant differences between teachers’ different age groups, in the proportions of teachers aged less than or over 60, or between full and associate professors compared with researchers and contract teachers.

No statistically significant differences were found concerning the technological, didactic, and emotional aspects related to DE in terms of the support provided or the support received in DE among teachers who had scores higher than the cut-off for depressive symptomatology compared to those who scored below the cut-off.

### Self-assessment of emotional condition perceived after the period of confinement

**Fig. 3:**
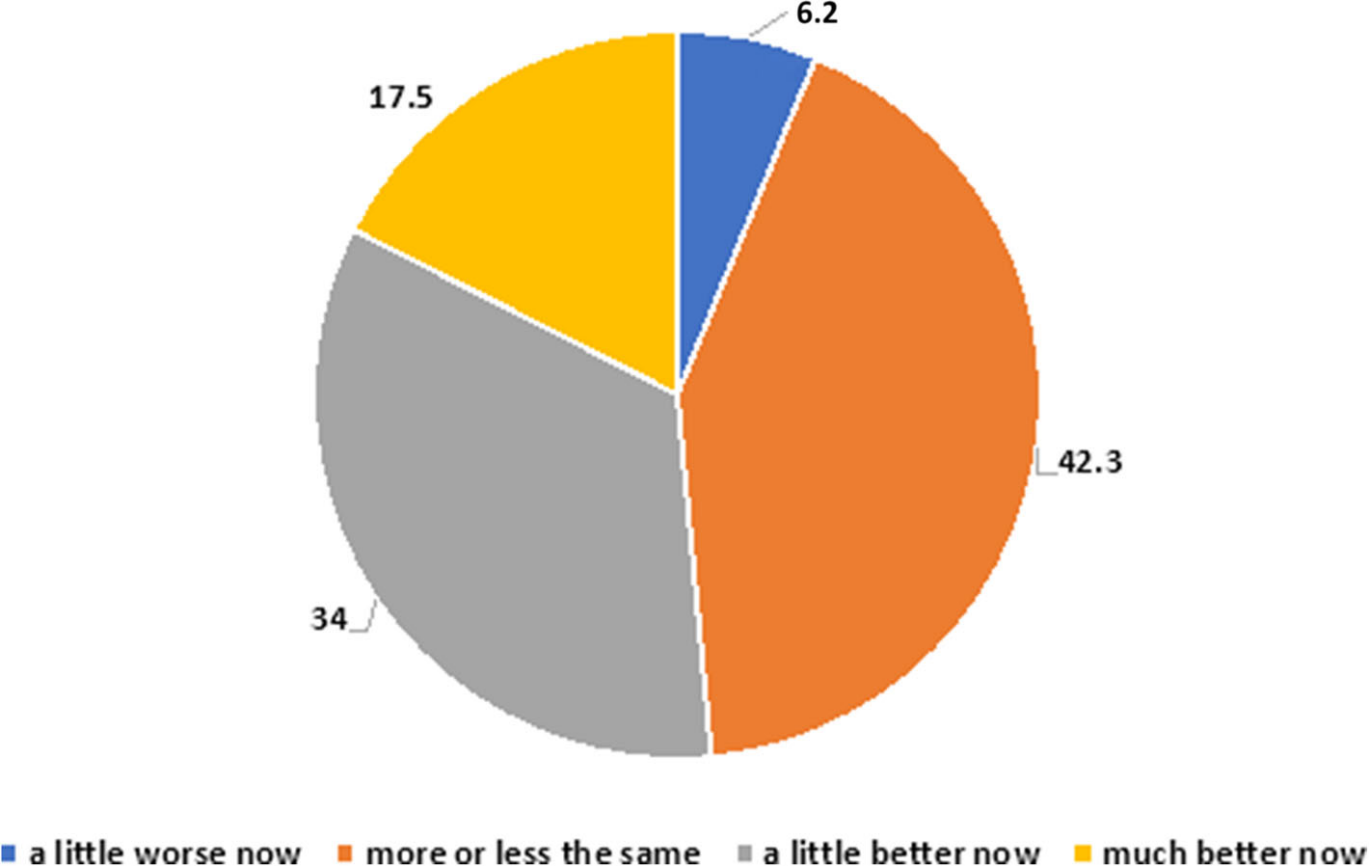
Self-assessment of emotional condition perceived after the period of confinement (*n* = 95)

Compared to the lockdown period, 42.3% of teachers (*n* = 41) who continued to deliver DE reported current relatively stable emotional well-being, whereas 17,5% (*n* = 17) reported greater well-being and 34% (*n* = 33) appreciated a slight improvement. Only 6 teachers (6.2%) expressed a worsening of their emotional condition (Fig. 3).

### Correlations between the overall evaluation of the experience of DE and the variables included in the study

The correlations between the score assigned by teachers to the overall evaluation of the DE experience, the age of the teachers, teaching hours in the second semester of 2020, advantages and disadvantages of DE, satisfaction with new learning, scores on the BDI-II referring to the period of confinement and the evaluation of emotional health perceived by the teachers at the end of the confinement period are shown in Table 7.

**Table 7:** Correlations among the overall evaluation of distance education (DE) experience, DE aspects, age of teachers, teaching hours in the second semester AY 2019–20, BDI-II scores during confinement and emotional health after confinement (*n* = 97)

		Overall evaluation of the DE experience	Teacher age	Teaching hours during the II semester AY 2019–20	Distance education advantages	Personal satisfaction with new learning	Distance education disadvantages	BDI-II scores during home confinement	Emotional health after confinement
**Teacher age**	Pearson’s Correlation	−.041	1						
	2-tailed *p*-value	.688							
**Teaching hours during the II semester AY 2019–20**	Pearson’s Correlation	−.153	.107	1					
	2-tailed p-value	.135	.296					.	
**Distance education advantages**	Pearson’s Correlation	.400^**^	.105	.059	1				
	2-tailed p-value	.000	.365	.614					
**Personal satisfaction with new learning**	Pearson’s Correlation	.752^**^	.006	−.120	.287^*^	1			
	2-tailed p-value	.000	.956	.281	.021				
**Distance education disadvantages**	Pearson’s Correlation	−.555^**^	.049	.214^*^	−.223	−.414^**^	1		
	2-tailed p-value	.000	.632	.035	.052	.000			
**BDI-II scores during home confinement**	Pearson’s Correlation	−.289^**^	−.070	.157	−.195	−.197	.172	1	
	2-tailed p-value	.005	.501	.130	.096	.079	.098		
**Emotional health after confinement**	Pearson’s Correlation	−.160	−.163	−.069	−.180^*^	−.207	.098	.344^**^	1
	2-tailed p-value	.117	.110	.500	.131	.060	.342	.001	

**p < 0.01

*p < 0.05

The good overall evaluation of DE was positively correlated with the advantages that the teachers identified and with satisfaction with the new learning stimulated by this teaching method. Furthermore, the overall evaluation of the teaching experience was significantly inversely correlated with the number of DE disadvantages and with BDI-II scores suggestive of depressive symptomatology during the lockdown, suggesting an association between a lower evaluation of DE, greater weight assigned to negative aspects and a high expression of depressive symptoms.

The number of disadvantages identified by the teachers was positively correlated with a high teaching load in hours during the second semester and inversely correlated with satisfaction with new learning.

Referring to the period of confinement, the BDI-II scores were statistically significantly positively correlated with the self-assessment of current emotional health after confinement, highlighting an “emotional well-being recovery” given that the highest scores for depression during lockdown were related to higher well-being scores after that period.

## Discussion

This study examined the experience of DE in a sample of university teachers during the second semester of the AY 2019–20. It highlights how the teaching staff coped technically and emotionally with the challenge of adapting to a new teaching method during the Italian lockdown period.

The urgent, imperative DE caused by the COVID-19 pandemic added to the stresses and workloads of university staff who were already struggling to balance teaching, research, and care obligations in addition to work-life balance [27, 28]. As in many countries, teaching staff of all backgrounds and ages had to prepare and deliver lessons from home, with all the practical and technical challenges that this entails and often without adequate technical support [2, 29].

Our survey showed a good response rate by the department’s permanent teachers, which exceeded our expectations given the lack of teachers’ practice in self-assessment.

Our teachers, with a rather high average age, responded promptly although almost half of the sample faced technical difficulties, such as those related to the quality of connections and family overlap in the use of the network and hardware, which were common problems among the general population in the period considered. It is interesting that our survey allowed us to grasp so many expected aspects, such as the effort employed in adopting an unfamiliar teaching methodology, the use of personal IT tools that cannot guarantee good presentations with the need to purchase more adequate tools at their own expense, and inconvenience due to the overlapping of their own needs for use with those of other family members in settings not always suited to the “sacredness” of the academic lesson.

About the challenges of emergency remote teaching strategy, our results confirmed the findings of Akour [19], reporting teachers complained about what we called the didactic aspect of DE, i.e., the amount of time and the effort needed to design examinations, registered also by Elsalem et al. [30]. The majority of that sample was concerned about the increased possibility of cheating during online distance examination, our teachers reported a lower impact of such issue, referred to their commitment to the supervision of oral and written examinations. On the psychological aspects, related to the intrusion of the teachers’ privacy and “contact at any time of the day irrespective to private personal time,” negatively perceived by more than half of Jordan teachers, our academics showed a different perspective, less concerned about their privacy, complained only by less than a quarter of them. In the Italian sample, the teacher’s closer and continuous contact with the students outside the lesson time, which was unthinkable before the lockdown, was reported as a positive aspect of DE by more than a third of the teachers, presumably appreciating the less formal relationship teacher-student created by the COVID-19 climate, stimulating a “sense of team” to cope against a traumatic event affecting the whole community. In our sample the most distressing psychological aspect was represented by the burden of speaking “to the void” in front of a screen.

When balancing the advantages and disadvantages of DE in our sample, the latter were more represented, especially concerning the absence of direct “face-to-face” contact, the difficulty of assessing students’ degree of attention, reported by the majority of teachers, that we can synthesize as the regret about the lack of energizing relationships with students in the classroom.

The lack [31] or the reduced [19] interaction with the students, the missing non-verbal communication [32], the impossibility of face-to-face teaching [22, 31], and verify if they understand the material [33] represent common issues underlining all the literature on DE, and our findings seemed to confirm the importance of face-to-face education. Moreover, especially for medical and health professions courses, among the DE disadvantages identified by our sample the impossibility of conducting specific interactive teaching methods and attendance of professional laboratories was reported, confirming Gaur et al. [34] concerns about the lack of “hands-on training”, which could have serious implications on the training of the current cohort of students.

Finally, a little-studied aspect in the literature was related to the emotional resonance with which the teacher faced the new, for some, anxious experience of DE, a mandatory new teaching methodology approached without adequate pedagogical preparation. The rate of depressive symptoms with different severity levels showed by a quarter of teachers of our sample was lower than that found by Akour [19], reporting that respectively one-third of Jordan teachers had severe distress and one-third had mild to moderate distress. But, if we consider the significant impairment of sleep patterns in more than half of our teachers, with loss of energy and greater difficulty concentrating reported in female teachers, our results are close to the findings of Akour [19]. These conditions express a state of distress, which must be interpreted as related not only to the discomforts of DE, but also to suffering from confinement and social isolation.

A satisfactory condition of well-being was linked to the perception of positive aspects of the DE experience and the new learning stimulated by this teaching method, as a protective factor against depression symptoms, that we could compare to “motivation for shifting the educational process into DE” of Akour et al. [19]. The overall evaluation of the DE experience of our teachers (scoring higher than 6 = 54.7%) reflects a lower motivation compared to the Jordan colleagues (moderate = 45.8%; strong/high motivation = 27%). The didactic load of the semester seems to have influenced the perception of disadvantages, contributing to a lower appreciation of the challenges of DE. The data show that greater manifestation of depressive symptoms during the lockdown predicted a greater subjective recovery of a good emotional condition after domestic confinement, characterized by greater individual freedom and a better social life, despite the maintenance of DE management. Undoubtedly, domestic confinement was experienced as a stressful event for both social life, and work and the provision of DE. Moreover, it is possible that the improvement in emotional state was due not only to the end of confinement but also to the teachers’ greater familiarity with digital technology.

Some teachers of our department, such as those at the University of L’Aquila, had already successfully faced the challenge following the disastrous earthquake of 2009 [23]. DE was not adopted 11 years ago. The time was not ripe, and the type of catastrophic event did not allow for its organization. We can hypothesize that our teachers, at least the older ones, from the traumatic experience of the L’Aquila 2009 earthquake learned the peer support, as registered by the specific section “given/received support” to assess the colleagueship, showing that more than half of teachers were supported by their colleagues in conducting DE, with a very low unsatisfaction in this area.

Currently, the introduction of DE in Italian universities has represented a valuable opportunity to pay more attention to university teaching and training themes. The school sector has absorbed the prevailing interest of the mass media and neglected attention to the university. In the present historical moment, state universities, which have always looked at telematic universities with an attitude of sufficiency, have been forced to impose on teachers and therefore on students the transitory but radical abandonment of teaching “in presence”, and now have to consider also its impact on teachers and students to optimize the enhanced teaching and learning practices in the post-digital era [21]. The COVID-19 pandemic could be seen not as a radical modifier but just an accelerator of the process of change of didactic [35, 36], or, better, a desirable integration to teaching modalities aimed to improve successful learning.

### Strengths and limitations

The main study strength is that it is the first Italian study evaluating the emotional resonance with which the university teacher faced the new DE experience during the COVID-19.

Some limitations of the present study should be acknowledged. Firstly, the study was conducted among a sample at a single academic department, and the sample size (of both participants and setting) represents a limitation for generalizability. Secondly, no validated measures were used in our study in the absence of international validated instruments assessing the DE. The “Teaching with the COVID 19” was developed by a specific focus group of experienced teachers, starting from their suggested main themes. All the teachers stated that they had to build new presentations, more accurately, considering the lack of interaction with students and their real involvement, since all lessons were recorded or uploaded by the teachers. The comparison of DE lessons with the “face-to-face” lessons was implicitly clear to all participants, despite the absence of data to compare the new DE workload with the previous perceived one. Thirdly, a more comprehensive battery for psychopathological assessment would have been useful for better characterize the teachers’ emotional problems. The aim was to investigate the presence of emotional suffering in teachers, not identifying a psychiatric diagnosis. Our short version of BDI reflects the Authors choice of being “less invasive” (items on suicide ideation, sexuality, etc., were omitted) and “normalizing” the sort of supposed post-traumatic reaction expected to follow to COVID-19 social restriction measures. Based on the experience of working with the post-traumatic distress of some of the Authors [37, 38] within certain limits, distress is perfectly natural and normal in such a situation as in the context of COVID-19 pandemic. Fourthly, we can hypothesize an answer bias, i.e., the respondents were more distressed than non-responders.

## Conclusions

Referred to the more rigorous period of Italian lockdown, as a security measure adopted to contain the COVID-19 pandemic, our study highlights the impact of technical, didactic, and psychological difficulties of DE, its advantages and disadvantages reported by our department university teachers. They showed a various level of emotional distress, as depressive symptoms, sleep problems, loss of energy, difficulty in concentration, the latter especially in female teachers. In our sample, teachers showed a good colleagueship, supporting each other in the challenge of DE. The appreciation of their new learning promoted by DE seemed related to better emotional well-being. The most substantial disadvantage complained was the lack of relational and reflective exchanges with students “in the classroom,” since teaching does not imply to transmit only “knowledge,” but also emotions, feelings, relationships, and positive memories in which the act of learning takes place. The greater subjective recovery of more severe depressed teachers once the domestic confinement was over, despite maintaining DE, could suggest a more comprehensive psychosocial traumatic model in the COVID-19 pandemic. In Italy, the confinement measures are still on, and it is not easily predictable when schools and universities will restart their routine activities. Beyond the larger availability of technical devices and increased familiarity with acquired DE skills, psychological support strategies should be planned to overcome persistent academic distress. Special attention should be given to university teachers considering their important role in the high-education system, influencing good learning and promoting student professional success.

## Supplementary Information

**Additional file 1.** Questionnaire on teachers perspectives on distance education (DE).

## Data Availability

The datasets used and/or analyzed the current study are available from the corresponding author on reasonable request.

## References

[1] World Health Organization, W (2020). Coronavirus disease (COVID-19) pandemic.

[2] Sahu P. Closure of Universities Due to Coronavirus Disease 2019 (COVID-19): Impact on Education and Mental Health of Students and Academic Staff. Review Cureus. 2020;12(4):e7541. 10.7759/cureus.7541. 10.7759/cureus.7541 PMC7198094 32377489

[3] Moore MG, Kearsley G (2011). Distance Education: A Systems View of Online Learning.

[4] Tarafdar M, Tu Q, Ragu-Nathan TS, Ragu-Nathan BS (2011). Crossing to the dark side: examining creators, outcomes, and inhibitors of technostress. Commun ACM.

[5] Qi C (2019). A double-edged sword? Exploring the impact of students’ academic usage of mobile devices on technostress and academic performance. Behav Inform Technol.

[6] Ayyagari R, Grover V, Purvis R (2011). Technostress: technological antecedents and implications. MIS Q.

[7] Tarafdar M, Tu QA, Ragu-Nathan TS (2010). Impact of technostress on end-user satisfaction and performance. J Manag Inf Syst.

[8] Dragano N, Lunau T (2020). Technostress at work and mental health: concepts and research results. Curr Opin Psychiatry.

[9] La Torre G (2019). Definition, symptoms and risk of techno-stress: a systematic review. Int Arch Occup Environ Health.

[10] Berg-Beckhoff G, Nielsen G, Ladekjaer Larsen E (2017). Use of information communication technology and stress, burnout, and mental health in older, middle-aged, and younger workers - results from a systematic review. Int J Occup Environ Health.

[11] Al-Fudail M, Mellar H (2008). Investigating teacher stress when using technology. Comput Educ.

[12] Jena RK (2015). Technostress in ICT enabled collaborative learning environment: an empirical study among Indian academician. Comput Hum Behav.

[13] Wang XH, Li B. Technostress Among University teachers in higher education: a study using multidimensional person-environment misfit theory. Front Psychol. 2019;10. 10.3389/fpsyg.2019.01791. 10.3389/fpsyg.2019.01791 PMC6691142 31447734

[14] Brooks SK, Webster RK, Smith LE, Woodland L, Wessely S, Greenberg N, Rubin GJ (2020). The psychological impact of quarantine and how to reduce it: rapid review of the evidence. Lancet.

[15] Xiang YT, Yang Y, Li W, Zhang L, Zhang Q, Cheung T, Ng CH (2020). Timely mental health care for the 2019 novel coronavirus outbreak is urgently needed. Lancet Psychiatry.

[16] Cao W, Fang Z, Hou G, Han M, Xu X, Dong J, Zheng J (2020). The psychological impact of the COVID-19 epidemic on college students in China. Psychiatry Res.

[17] Giusti L, et al. #Everythingwillbefine. Duration of home confinement and “all-or-nothing” cognitive thinking style as predictors of traumatic distress in young university students on a digital platform during the COVID-19 Italian lockdown. Front Psychiatry. 2020;11:574812. 10.3389/fpsyt.2020.574812. eCollection 2020. 10.3389/fpsyt.2020.574812 PMC7770221 33384623

[18] Odriozola-Gonzalez P (2020). Psychological effects of the COVID-19 outbreak and lockdown among students and workers of a Spanish university. Psychiatry Res.

[19] Akour A, al-Tammemi A’B, Barakat M, Kanj R, Fakhouri HN, Malkawi A, Musleh G (2020). The impact of the COVID-19 pandemic and emergency distance teaching on the psychological status of university teachers: a cross-sectional study in Jordan. Am J Trop Med Hyg.

[20] Ching Y-H, Hsu Y-C, Baldwin S (2018). Becoming an Online Teacher: An Analysis of Prospective Online Instructors’ Reflections. J Interact Learn Res.

[21] Rapanta C, Botturi L, Goodyear P, Guàrdia L, Koole M (2020). Online University teaching during and after the Covid-19 crisis: refocusing teacher presence and learning activity. Postdigital Sci Educ.

[22] Georgoulias P, et al. COVID-19 crisis: will online learning have negative consequences to our students? Cardiol Young. 2021;31(3):511. 10.1017/S104795112000493X. Epub 2021 Jan 7. 10.1017/S104795112000493X PMC7844167 33410732

[23] Casacchia M, Pollice R, Roncone R (2012). The narrative epidemiology of L'Aquila 2009 earthquake. Epidemiol Psychiatr Sci.

[24] Casacchia M, Bianchini V, Mazza M, Pollice R, Roncone R (2013). Acute stress reactions and associated factors in the help-seekers after the L'Aquila earthquake. Psychopathology.

[25] Roncone R, Giusti L, Mazza M, Bianchini V, Ussorio D, Pollice R, Casacchia M (2013). Persistent fear of aftershocks, impairment of working memory, and acute stress disorder predict post-traumatic stress disorder: 6-month follow-up of help seekers following the L'Aquila earthquake. Springerplus.

[26] Beck AT, Steer RA, Ball R, Ranieri WF (1996). Comparison of Beck depression inventories -IA and -II in psychiatric outpatients. J Pers Assess.

[27] Houlden S, Veletsianos G (2020). Coronavirus pushes universities to switch to online classes – but are they ready?.

[28] Houston D, Meyer LH, Paewai S (2006). Academic staff workloads and job satisfaction: expectations and values in academe. J High Educ Policy Manag.

[29] Hodges, C., et al. The difference between emergency remote teaching and online learning. Educ Rev. 2020. Available from: https://er.educause.edu/articles/2020/3/the-difference-between-emergency-remote-teaching-and-online-learning. Accessed 10 Dec 2020

[30] Elsalem L, al-Azzam N, Jum'ah AA, Obeidat N (2021). Remote E-exams during Covid-19 pandemic: A cross-sectional study of students’ preferences and academic dishonesty in faculties of medical sciences. Ann Med Surg (Lond).

[31] Hebebci MT, Bertiz Y, Alan S (2020). Investigation of views of students and teachers on distance education practices during the coronavirus (COVID-19) pandemic. Int J Technol Educ Sci.

[32] Knie K (2020). To zoom or not to zoom - the training of communicative competencies in times of Covid 19 at Witten/Herdecke University illustrated by the example of “sharing information”. GMS J Med Educ.

[33] Gewin V (2020). Five tips for moving teaching online as COVID-19 takes hold. Nature.

[34] Gaur U, et al. Challenges and Opportunities of Preclinical Medical Education: COVID-19 Crisis and Beyond. Review SN Compr Clin Med. 2020;1-6. 10.1007/s42399-020-00528-1. Online ahead of print. 10.1007/s42399-020-00528-1 PMC7508422 32984766

[35] Yakup A, Biçerb N, Canc F (2020). Perspectives of Pre-Service Teachers on Distance Education: Covıd-19 Process. Rev Argentina Clín Psicol.

[36] Tesar M (2020). Towards a post-Covid-19 ‘new normality?’: Physical and social distancing, the move to online and higher education. Policy Futures Educ.

[37] Hamblen JL, et al. La sofferenza psicologica da disastri naturali e traumi importanti. Trattamento cognitivo-comportamentale. Manuale per gli operatori. (Manual for professionals). Roma: Il Pensiero Scientifico Editore; 2018.

[38] Hamblen JL, et al. La sofferenza psicologica da disastri naturali e traumi importanti. Trattamento cognitivo-comportamentale. Quaderno di lavoro per l’utente. (Manual for users). Roma: Il Pensiero Scientifico Editore; 2018.

