# Supplementary material for: Distance education during COVID 19: an Italian survey on the university teachers’ perspectives and their emotional conditions
Source: BMC Med Educ. 2021 Jun 9;21:335. doi: 10.1186/s12909-021-02780-y (PMC8187887; doi:10.1186/s12909-021-02780-y)
Supplement: Supplementary file 1 — Additional file 1. Questionnaire on teachers perspectives on distance education (DE). [file 12909_2021_2780_MOESM1_ESM.docx]

**Appendix 1. QUESTIONNAIRE ON TEACHERS PERSPECTIVES ON DISTANCE EDUCATION (DE)**

**SECTION 1. - INFORMATION ON THE STUDY, PROTECTION OF PRIVACY, INFORMED CONSENT**

**SECTION 2. DISTANCE EDUCATION ON THE PART OF THE TEACHER**

**MAIN DEMOGRAPHIC AND ACADEMIC DATA**

**1. Sex**

- Man
- Woman

**2. Age [__] [__]**

**3. Academic role:**

- Full professor
- Associate professor
- Researcher
- Temporary researcher (TDB)
- Contract teacher
- Hospital contract teacher

**4.** **Select the Department course/courses you teach**

- Second-Level Degree (unique 6-year cycle) Course in Medicine and Surgery
- Second-Level Degree (unique 6-year cycle) in Dentistry and Dental Prosthetics
- Second-Level Degree in Nursing and Obstetric Sciences
- Second-Level Degree in Sciences of Technical Health Professions
- Second-Level Degree in Sciences of Prevention Health Professions
- First-Level Degree Course in Nursing
- First-Level Degree Course in Speech Therapy
- First-Level Degree Course in Obstetrics
- First-Level Degree Course in Orthoptics and Ophthalmological Assistance
- First-Level Degree Course in Psychiatric Rehabilitation Technique
- First-Level Degree Course in Neuro and Psychomotor Childhood Therapy
- First-Level Degree Course in Dental Hygiene
- First-Level Degree Course in Techniques of Prevention in the Environment and in the Workplace
- Second-Level Degree Course in Biology of Health and Nutrition
- First-Level Degree Course in Biological Sciences
- Second-Level Degree Course in Environmental Biology and Ecosystem Management
- First-Level Degree Course in Environmental Sciences and Technologies

**5. In the second semester A.Y. 2019-20, you held lessons in?**

- One course
- More course
- I held my lessons in the first semester
- Other (*specify ……………………………………….)*

**6.** **In the second semester A.Y. 2019-20, you also held lessons in master’s program?**

- No
- Yes

If Yes, *specify …………………………………………………………………………..*

**7.** **In the second semester A.Y. 2019-20, you also held lessons in other Departments?**

- No
- Yes

If Yes, *specify …………………………………………………………………………..*

**8.** **In the second semester A.Y. 2019-20, how many hours have you taught (add up all the teaching hours)?** [_][_][_]

- No
- Yes

**EXPERIENCE IN THE DELIVERY OF THE DIDACTIC MODUL THROUGH DISTANCE EDUCATION**

**DISTANCE EDUCATION EXPERIENCE**

**How was your experience concerning the TECHNOLOGICAL ASPECTS of DE in the second semester A.Y. 2019-2020?**

|  | **No difficulty** | **Occasional difficulties** | **Some difficulty** | **Many difficulties** | **Major difficulties** |
| --- | --- | --- | --- | --- | --- |
| **9.** Connection quality |  |  |  |  |  |
| **10.** Device availability |  |  |  |  |  |
| **11.** Use of TEAMS platform |  |  |  |  |  |
| **12.** Other |  |  |  |  |  |

**13. In your home, have you had problems with family overlap in the use of the network and hardware?**

- No problems
- Minor inconveniences, without significant consequences
- Significant inconvenience and frequent loss of effectiveness of the connection and / or alternation in hardware sharing
- Frequent inability to connect due to network or hardware sharing

**How was your experience concerning the DIDACTIC ASPECTS of DE in the second semester A.Y. 2019-2020?**

|  | **Major difficulties** | **Many difficulties** | **Some difficulty** | **Occasional difficulties** | **No difficulty** |
| --- | --- | --- | --- | --- | --- |
| **14.** Working time to organize and structure the materials of the lessons for DE |  |  |  |  |  |
| **15.** Working time to record lessons for DE |  |  |  |  |  |
| **16.** Supervision of oral examination |  |  |  |  |  |
| **17.** Supervision of written examination |  |  |  |  |  |
| **18.** Other |  |  |  |  |  |

**How was your experience concerning the PSYCHOLOGICAL ASPECTS of DE in the second semester A.Y. 2019-2020?**

|  | **Major difficulties** | **Many difficulties** | **Some difficulty** | **Occasional difficulties** | **No difficulty** |
| --- | --- | --- | --- | --- | --- |
| **19.** Speaking "in the void" through a camera, without face-to-face contact with the students |  |  |  |  |  |
| **20.** Accuracy of materials to be left on TEAMS platform to students |  |  |  |  |  |
| **21.** Acritical diffusion of uploaded materials by the students |  |  |  |  |  |
| **22.** Perception that students were only waiting for the uploaded materials and losing interest in the online lesson |  |  |  |  |  |
| **23.** Awareness of being recorded during the lesson |  |  |  |  |  |
| **24.** Awareness of being observed in one’s own private household |  |  |  |  |  |
| **25.** Other |  |  |  |  |  |

**ADVANTAGES OF DISTANCE EDUCATION**

**What positive aspects did you find in DE compared to the traditional teaching (multiple answers are possible)?:**

***Relationship with students***

- **26.** Greater involvement and attention of students during the lessons based on the questions addressed in the chat
- **27.** Greater personalized knowledge of students
- **28.** Greater availability of contacts outside class hours for clarification via email and WhatsApp
- **29.** Greater “sense of team” and collaboration between students and teachers, aimed at better preparation for exams
- **30.** Other (specify ……………………… ..)

***Didactic*** ***and organizational aspects***

- **31.** Greater care in adapting the lessons to stimulate the students and involve them in active participation
- **32.** Opportunity to record and upload lessons on the platform outside the canonical lesson times
- **33.** Greater respect for lesson times
- **34.** Reduction of the travel time required to reach the university, with the advantage of greater availability of time to devote to students and teaching activities (for nonresident teachers in L'Aquila)
- **35.** I honestly didn't find any positive aspects
- **36.** Other (specify ……………………………………… )

**In the period of confinement, in relation to the provision of the DE, were you able to "save" due to (multiple answers are possible)?**

- **37.** Missing fuel costs to reach the workplace
- **38.** Missed catering expenses
- **39.** Missed travel expenses to/from L'Aquila for routes longer than 20 km
- **40.** Missed living expenses in L'Aquila (any overnight stays at the workplace)
- **41.** I was not able to save
- **42.** *(For clinicians not resident in L'Aquila)* For my clinical activity, I continued to travel from / to L'Aquila
- **43.** Other (specify ………………………………….)

**44. If you were to estimate, at least briefly, the savings allowed by carrying out Distance Education, can you kindly indicate how much (in euros)? [__] [__] [__] [__]**

**Did you have opportunities to experience more frequent “virtual” exchanges with your Colleagues in Distance Education (multiple answers are possible)?**

- **45.** The coordinator of the integrated course^^[[1]](#footnote-1)^^ more easily promotes opportunities for discussion and collaboration between the teachers of the modules, included in the integrated course
- **46.** Semester coordinators are facilitated in the work of vertical and horizontal integration of the disciplines
- **47.** The teachers of the integrated course are facilitated in agreeing on the examination method and assessment
- **48.** Distance Education has not given me the opportunity to experience any opportunity in this field
- **49.** Other (specify …………………………………………………………….)

**What personal improvements have you noticed in relation to the provision of DE (multiple answers are possible)?**

- **50.** Improvement in power point lesson presentations (animations, video file insertion, transformation into .mp4 file, etc.)
- **51.** Learning from scratch - improvement in the use of platforms
- **52.** Learning from scratch - improvement in the use of platforms / software for teaching
- **53.** Learning from scratch - improvement in the use platforms / software for conducting online exams
- **54.** I have not learned anything new
- **55.** Other (specify ……………………… ..)

**56. How satisfied are you with your new personal learning stimulated by DE (improvement of presentations and materials, use of digital platforms)?** (1 = not at all satisfied; 10 = very satisfied) **[__] [__]**

**DISADVANTAGES OF DISTANCE EDUCATION**

**What negative aspects did you find in DE compared to the traditional teaching (multiple answers are possible)?:**

***Relationship with students***

- **57.** Absence of direct "face-to-face" eye contact.
- **58.** Difficulty of assessing students’ degree of attention
- **59**. Reduced interaction with students during lessons
- **60.** Distracting effect due to the students’ study environment.
- **61.** I didn't find any negative aspects
- **62.** Other (specify ………………………………….)

***Didactic and organizational aspects***

- **63.** Impossibility of conducting specific teaching and training methods (e.g., work in small groups, role play)
- **64.** Impossibility of organizing laboratory professionalizing activities
- **65.** Difficulty of receiving students
- **66.** Difficulties related to carrying out small administrative procedures related to teaching (i.e, register Course of Other Training Activities, Optional Training Activites, ect.…)
- **67.** I didn't find any negative aspects
- **68.** Other (specify ………………………………….)

**69. What additional expenses did you incur?**

- Telephone and network consumption
- Costs for upgrading the home network
- Purchase of a laptop
- Purchase desktops
- Purchase consumables (cartridges, paper, etc.)
- Purchase headphones / microphones
- Purchase a camcorder
- Costs for purchase / usage fees for software for DE
- Costs for consultancy / repairs, IT adaptations
- I have not incurred any additional expenses
- Other (specify ............)

**70. If you should, at least briefly, estimate the costs incurred personally for the development of Distance Education, can you kindly indicate how much you spent (in euros)? [__] [__] [__] [__]**

**SUPPORT PROVIDED TO COLLEAGUES IN DIFFICULTY/SUPPORT RECEIVED BY COLLEAGUES**

**For your role as President and / or Director of the Degree Course, or for your greater experience as a teacher, in relation to the provision of DE (multiple answers are possible)?:**

- **71.** I helped colleagues in the new planning of teaching activities
- **72.** I helped my colleagues with the practical aspects related to the Teams platform
- **73.** I helped colleagues in carrying out the DE activity
- **74.** I helped Colleagues in maintaining contact with other teachers
- **75.** I helped Colleagues in maintaining contact with students
- **76.** I did not received any request
- **77.** Other (specify ……………………)

**In the provision of Distance Education**

- **78**. I was helped by my colleagues in the new planning of the teaching activity
- **79.** I was supported by my colleagues with the practical aspects related to the Teams platform
- **80.** My colleagues helped me in carrying out the DE activity
- **81.** My colleagues helped me in maintaining contact with other teachers
- **82.** I was assisted by my colleagues in maintaining contact with students
- **83.** I didn't need any support
- **84.** Although I wanted to be helped, I found no support
- **85.** Other (specify ………………….)

**OVERALL EVALUATION OF THE DE EXPERIENCE**

**86. How do you assess the DE experience overall (1 = very negative; 10 = excellent)? [_][_]**

**87. Comments and suggestions about DE**

…………………………………………………………………………………………………………………………………………………………

…………………………………………………………………………………………………………………………………………..……………

……………………………………………………………………………………………………………………………………………………..…

**SECTION 3.** **THE TEACHER'S EMOTIONAL WELL-BEING DURING COVID-19 LOCKDOWN (OPTIONAL COMPILATION)**

*In the 2 months of confinement due to COVID-19, it is possible that, as a person, you have had moments of difficulty and emotional distress. We would be grateful if you could answer the questions presented to you below, which will help us understand how much your state has influenced any perception of difficulty and dissatisfaction, even in your role as a teacher. In answering the questions, we invite you to refer to the 2 months of confinement (mid-March - early May 2020).*

**88. Sadness**

0. I did not feel sad

1. I felt sad

2. I was sad all the time and I couldn't snap out of it

3. I was so sad or unhappy that I couldn't stand it

**89. Pessimism**

0. I was not particularly discouraged about the future

1. I felt discouraged about the future

2. I felt I had nothing to look forward to

3. I felt the future was hopeless and that things could not improve

**90. Loss of pleasure**

0. I got as much satisfaction out of things as I used to

1. I didn’t enjoy things the way I used to

2. I didn’t get real satisfaction out of anything anymore

3. I was dissatisfied or bored with everything

**91. Loss of interest**

0. I didn't lose interest in other people or activities

1. I was less interested in other people or things than before

2. I lost most of my interest in other people or things

3. It was hard to get interested in anything

**92. Loss of energy**

0. I had as much energy as ever

1. I had less energy than I used to have

2. I didn't have enough energy to do very much

3. I didn't have enough energy to do anything

**93. Changes in sleeping pattern**

0. I did not experience any change in my sleeping pattern

1.a I slept somewhat more than usual

1.b I slept somewhat less than usual

2.a I slept a lot more than usual

2.b I slept a lot less than usual

3.a I slept most of the day

3.b I woke up 1-2 hours early and I couldn’t get back to sleep

**94. Irritability**

0. I was not more irritable than usual

1. I was more irritable than usual

2. I was much more irritable than usual

3. I was irritable all the time

**95. Changes in appetite**

0. I did not experience any change in my appetite

1.a My appetite was somewhat less than usual

1.b My appetite was somewhat greater than usual

2.a My appetite was much less than usual

2.b My appetite was much greater than usual

3.a I had no appetite at all

3.b I craved food all the time

**96. Concentration difficulty**

0. I could concentrate as well as ever

1. I couldn’t concentrate as well as usual

2. It was hard to keep my mind on anything for very long

3. I found I couldn't concentrate on anything

**97. Tiredness or fatigue**

0. I was no more tired or fatigued than usual

1. I got tired or fatigued more easily than usual

2. I was too tired or fatigued to do a lot of the things I used to do

3. I was too tired or fatigued to do most of the things I used to do

**98.Compared to the beginning of last May (end of confinement), how would you judge your emotional condition now?**

5 = much better now

4 = a little better now

3 = more or less the same

2 = a little worse now

1 = much worse now

*Thanks for your collaboration*

1. In the Italian University degree courses, the Coordinator of an Integrated Course exercises the following functions: coordinates the didactic programs concerning the objectives of the integrated course itself; represents the reference figure of the Course for the students; coordinates the preparation of the exams. He usually chairs the Examination Commission of the Course coordinated by him and proposes its composition. He is responsible for the proper conduct of all educational activities required for achieving the objectives defined for the course itself and the proper conduct of the examination tests [↑](#footnote-ref-1)
